# Information-Geometric Detection via Local SPD Structure Fields in the Time–Frequency Domain

**DOI:** 10.3390/e28060679

**Published:** 2026-06-12

**Authors:** Yaohao Yue, Benjie Wei, Yang Yang

**Affiliations:** 1 School of Information Science and Engineering, Shandong University, Qingdao 266237, China; yaohaoy@mail.sdu.edu.cn; 2 National Space Science Center, Chinese Academy of Sciences, Beijing 100190, China

**Keywords:** information geometry, SPD manifold, affine-invariant Riemannian metric, local structure field, signal detection, fixed false-alarm probability, time–frequency analysis

## Abstract

Non-stationary signal detection is challenging when discriminative information is not reflected in global energy, mean spectra, or a single covariance statistic, but is instead embedded in the organization of local time–frequency structures. This paper proposes an information-geometric detector defined on local symmetric positive definite (SPD) structure fields. Time–frequency patches are transformed into a spatially distributed field of second-order tensors to characterize local directional organization and anisotropy. Under a locally isotropic Riemannian Gaussian approximation on the SPD manifold, the local distance-difference evidence is monotonically related to an approximate log-likelihood ratio, providing an information-geometric interpretation without implying strict Neyman–Pearson optimality. Instead of forming a single global statistic or stacking patch-level features, the proposed method constructs a spatially distributed field of structured SPD objects and derives local distance-difference evidence, which is subsequently aggregated into a sample-level detection statistic. Experiments under a controlled SPD structure-field locality benchmark show that performance gains are primarily driven by the proposed SPD structure-field representation, with the Riemannian metric providing only secondary refinement.

## 1. Introduction

Non-stationary signal detection is a fundamental problem in statistical signal processing and arises widely in radar sensing and wireless communications. Classical detection theory is usually formulated within the framework of binary hypothesis testing and the likelihood-ratio test, and detection performance is commonly evaluated under fixed false-alarm probability constraints [1]. However, many traditional detection statistics rely on global energy, mean spectral features, template correlation, or a single covariance matrix. When the two classes have similar global low-order statistics, discriminative information may be carried primarily by local time–frequency structures, directional organization, and spatial arrangement. In such cases, global statistics may suppress or discard the local evidence that is most relevant to detection.

Time–frequency analysis provides a finer representation for non-stationary signals and can reveal structural phenomena such as transient energy concentration, local ridges, frequency drift, and fragmented textures [2,3]. Existing spectro-temporal patch methods have shown that structural patterns within local time–frequency patches can provide effective information for language identification and acoustic event detection [4,5,6]. Nevertheless, most of these methods still treat spectrogram patches as ordinary intensity maps, template vectors, or inputs to learning models, and they lack explicit geometric modeling of local directional relationships and anisotropy. By contrast, the structure tensor can stably characterize directional energy, directional coupling, and anisotropy through local second-order gradient statistics [7,8,9]. After regularization, it naturally forms a symmetric positive definite (SPD) matrix object. Therefore, transforming a time–frequency patch into an SPD-valued structure field provides a more appropriate representation for detection tasks dominated by local structural information.

In this work, an SPD field is a spatially distributed collection of local symmetric positive definite tensors. Each tensor describes the local directional organization and anisotropy of a time–frequency patch, while the field preserves how such local structures are spatially arranged across the entire observation.

For the purpose of this study, the relevant non-stationary behavior is considered from a structural time–frequency perspective. The signal characteristics of interest are not assumed to be fully described by a time-invariant spectrum, a stationary covariance model, or a global energy statistic. Instead, the class-relevant information may appear as local organization patterns in the time–frequency plane, including local ridges, drifting spectral components, directional continuity, fragmented structures, or spatially localized texture changes. The purpose of introducing a local SPD structure field is therefore to model how these local time–frequency structures are organized, rather than to detect non-stationarity merely as a binary property of a signal.

The SPD manifold and information geometry provide intrinsic metrics for comparing such structural objects [10,11,12,13,14]. Previous studies have shown that the affine-invariant Riemannian metric (AIRM), the Karcher mean, the Riemannian geometric mean, and related matrix-manifold methods have been used for covariance modeling and brain–computer interface classification [15,16]. In radar detection, information-geometric and matrix information-geometric methods usually represent an observation cell as a single covariance matrix, Hermitian positive definite (HPD) matrix, or cell-level matrix object, and then construct detection statistics using matrix distances, divergences, or geometric filtering [17,18,19,20,21,22,23]. These methods effectively exploit the non-Euclidean geometry of matrix objects, but most remain at the level of sample-wise or cell-level matrix comparison and preserve little of the spatial distribution of local structures inside a patch.

Recent matrix-manifold studies further show that SPD/HPD matrix representations can be equipped with different intrinsic metrics, alignment operations, and discriminative projections. For example, O(n)-invariant Riemannian metrics provide a broad geometric framework for SPD matrices, Riemannian Procrustes analysis uses geometry-aware transformations for transfer learning on SPD covariance features, and discriminative HPD-manifold projection has been used for matrix information-geometric radar detection in non-Gaussian clutter [24,25,26]. These works reinforce the importance of respecting matrix-manifold geometry when positive-definite matrix objects are used as signal representations.

This leaves a methodological gap. Time–frequency patch methods preserve locality but usually lack SPD geometry-consistent modeling; SPD information-geometric detectors have a mature manifold foundation but often compress each observation into a single matrix object; and structure-tensor methods describe local directional organization but have rarely been formulated as sample-level detection statistics under fixed false-alarm probability ($P_{\mathrm{fa}}$) constraints. The present work addresses this gap by modeling each sample as a spatially distributed local SPD structure field, thereby preserving both the local organization of time–frequency structures and the intrinsic geometry of SPD matrices.

Motivated by this gap, this paper proposes an information-geometric detector based on local SPD structure fields for non-stationary signal detection problems in which the discriminative information is mainly reflected in local time–frequency structural organization. The proposed method first represents a time–frequency log-amplitude patch as a local SPD structure field composed of pointwise structure tensors. It then estimates two class-conditional pointwise Karcher mean reference fields under AIRM, and constructs local distance-difference evidence using the pointwise difference between squared AIRM geodesic distances. Finally, discriminative weighting, block-wise robust evidence pooling, and fixed-$P_{\mathrm{fa}}$ threshold calibration based on an independent null-hypothesis ($H_{0}$) calibration set are combined to form a sample-level detection statistic.

The main contributions of this paper are fourfold. First, we introduce an object-level reformulation for non-stationary signal detection by using local symmetric positive definite (SPD) structure fields as detection objects. This reformulation bridges time–frequency patch representations and Riemannian matrix-based detection frameworks, shifting the detection object from conventional scalar statistics or single-matrix summaries to spatially distributed structural objects.

Second, we construct a geometry-consistent relative-closeness detector on the proposed SPD structure-field representation. Class-conditional reference fields are estimated by pointwise Karcher means under the affine-invariant Riemannian metric (AIRM), and local distance-difference evidence is defined through pointwise differences between squared AIRM geodesic distances. This provides a field-level, geometry-consistent reformulation of the classical relative-closeness decision principle on the SPD manifold.

Third, we develop a robust evidence aggregation mechanism for local-structure-dominated detection. Discriminative weighting is used to emphasize stable local structural regions, whereas block-wise robust pooling suppresses low-information areas, local misalignment, and abnormal responses. The resulting aggregation framework integrates spatially nonuniform local distance-difference evidence into a sample-level detection statistic under fixed-$P_{\mathrm{fa}}$ calibration.

Fourth, we provide a mechanism-level analysis and validation framework based on a controlled SPD-field structured-locality benchmark, baseline comparisons, ablation studies, structural perturbation experiments, non-Gaussian background experiments, and paired-difference analysis. This analysis separates the roles of the object-layer representation and the Riemannian metric, showing that the local SPD structure-field representation is the primary source of performance gain, while Riemannian geometry provides a complementary consistency constraint and improves stable comparison within the same representation layer.

The remainder of this paper is organized as follows. Section 2 introduces the theoretical motivation and algorithmic construction of the proposed detector. Section 3 presents the experimental setup and results. Section 4 discusses the methodological relationships, applicability limits, and future directions. Section 5 concludes the paper.

## 2. Materials and Methods

### 2.1. Problem Formulation and Information-Geometric Motivation

Classical binary hypothesis testing is usually formulated within the likelihood-ratio test (LRT) framework [1]. Under an ideal setting in which the statistical models are known, let the observation *x* follow the probability models $p_{0}\left(x\right)$ and $p_{1}\left(x\right)$ under the null hypothesis $H_{0}$ and the alternative hypothesis $H_{1}$, respectively. The classical likelihood-ratio test can then be written as

(1) $$\Lambda \left(x\right)=\frac{p_{1}\left(x\right)}{p_{0}\left(x\right)}\overset{H_{1}}{\underset{H_{0}}{≷}}\eta ,$$

where η is the decision threshold. For *N* independent and identically distributed observations ${\left\{x_{i}\right\}}_{i=1}^{N}$, the normalized log-likelihood ratio is

(2) $$\frac{1}{N}\sum_{i=1}^{N}\ln\Lambda \left(x_{i}\right)=\frac{1}{N}\sum_{i=1}^{N}\ln\frac{p_{1}\left(x_{i}\right)}{p_{0}\left(x_{i}\right)}.$$

As N→∞, the law of large numbers implies that the above expression converges to an expectation under the true distribution q(x). Combined with the Kullback–Leibler (KL) divergence [27],

(3) $$D\left(q∥p\right)=\int q\left(x\right)\ln\frac{q(x)}{p(x)}\;dx,$$

the normalized log-likelihood ratio is equivalent to

(4) $$D\left(q∥p_{0}\right)-D\left(q∥p_{1}\right).$$

Therefore, under ideal large-sample conditions, classical detection can be interpreted as a relative-closeness decision in the sense of KL divergence [18,28]: the observation distribution is compared with the two hypothesized models, and the class with the smaller Kullback–Leibler divergence is selected.

This interpretation is used only as theoretical motivation. In the present work, the detection object is not a distribution-level hypothesis model or a single global covariance matrix, but an SPD-valued structure field defined over a local support Ω:

(5) $$S=\left\{S\left(x\right):x\in \Omega \right\},\;S\left(x\right)\in S_{++}^{2}.$$

Here, $S_{++}^{2}$ denotes the set of 2×2 symmetric positive definite matrices.

Correspondingly, the objects to be compared are no longer the two probability models $p_{0}$ and $p_{1}$, but two class-conditional reference structure fields,

(6) $$S_{0}=\left\{S_{0}\left(x\right):x\in \Omega \right\},\;S_{1}=\left\{S_{1}\left(x\right):x\in \Omega \right\}.$$

This object-layer redefinition is the key starting point that distinguishes the proposed method from the classical detection object level.

On this new object layer, the idea of classical relative closeness can be reformulated at the field level. Let $d_{R}\left(\cdot ,\cdot \right)$ denote the geodesic distance on the SPD manifold, and let w(x)≥0 be a spatial weight. The field-level energy functional relative to the class-*c* reference structure field can be defined as

(7) $$E_{c}\left(S\right)=\sum_{x\in \Omega }w\left(x\right)d_{R}^{2}\left(S\left(x\right),S_{c}\left(x\right)\right),\;c\in \left\{0,1\right\}.$$

The corresponding field-level relative-closeness statistic is then defined as

(8) $$T_{\mathrm{geo}}\left(S\right)=E_{0}\left(S\right)-E_{1}\left(S\right).$$

A larger value of $T_{\mathrm{geo}}\left(S\right)$ indicates that the test structure field is globally closer, in the geometric sense, to $S_{1}$ than to $S_{0}$; conversely, a smaller value indicates greater closeness to $S_{0}$. Expanding Equation (7) gives

(9) $$T_{\mathrm{geo}}\left(S\right)=\sum_{x\in \Omega }w\left(x\right)\left[d_{R}^{2}\left(S\left(x\right),S_{0}\left(x\right)\right)-d_{R}^{2}\left(S\left(x\right),S_{1}\left(x\right)\right)\right].$$

This expression shows that field-level relative closeness can be naturally written as a spatially weighted sum of pointwise differences between squared geodesic distances. The local distance-difference evidence and its sample-level aggregation developed below are constructed precisely from this field-level reformulation.

The field-level statistic above is used only to explain the design logic of the proposed method, not as a formal optimality statement for the proposed detector. The proposed method inherits the geometric idea of making a decision according to relative closeness from classical detection [29,30,31], but on the new detection object of local SPD structure fields, it redefines the class-conditional reference centers, the local comparison criterion, and the sample-level evidence aggregation mechanism.

### 2.2. Local Time–Frequency Structure-Field Representation

Let the observed discrete-time signal be x[n], where n=0,1,…,N−1. To characterize local structural features of non-stationary signals in the joint time–frequency domain, we first use the short-time Fourier transform (STFT) to map the raw observation onto the time–frequency plane [2,3]. It is defined as

(10) $$X\left(m,k\right)=\sum_{n=0}^{N-1}x\left[n\right]\omega \left[n-mR\right]e^{-j2\pi kn/K},$$

where ω[·] is the analysis window, *R* is the frame shift, *m* is the time-frame index, *k* is the frequency-bin index, and *K* is the number of discrete frequency points. The corresponding magnitude spectrum is denoted by

(11) A(m,k)=|X(m,k)|.

In this paper, the time–frequency representation is not the final decision object, but an intermediate domain for constructing the local structure-field representation. Compared with the raw one-dimensional waveform, the time–frequency representation can more directly reveal transient energy concentration, local ridges, directional continuity, frequency drift, and fragmented textures. These local organizational patterns are often more directly related to the task-relevant discriminative information in complex non-stationary detection tasks than any single global statistic.

Thus, the role of non-stationarity in the proposed pipeline is twofold. First, it motivates the use of a time–frequency representation, because the discriminative structures may change with time and may not be visible in a global spectrum or in raw waveform energy alone. Second, it motivates a local field representation rather than a single pooled statistic, because the useful evidence may be spatially localized, directionally organized, and unevenly distributed over the time–frequency support. The subsequent SPD structure-field construction is designed to preserve this local organization at the object level.

To reduce numerical instability caused by a large amplitude dynamic range and to enhance the separability of weak structural regions, we further use the log-amplitude representation

(12) $$L\left(m,k\right)=\log\left(A\left(m,k\right)+\varepsilon_{\alpha }\right),$$

where $\varepsilon_{\alpha }>0$ is a small regularization term used to avoid numerical singularities. Local patches are then extracted from L(m,k), and the patch support is denoted by

(13) $$\Omega \subset Z^{2},\;x=\left(t,f\right)\in \Omega .$$

The input object of the subsequent detector is the local structure field defined on Ω. This object choice means that the focus of the paper is not the intensity values themselves, but the directional relationships and spatial organization of local structural units within the patch.

On a local patch, we first compute the local gradient of the log-amplitude map,

(14) $$\nabla L\left(x\right)=\left[\begin{array}{c} L_{t}\left(x\right)\\ L_{f}\left(x\right) \end{array}\right],\;x\in \Omega ,$$

where $L_{t}\left(x\right)$ and $L_{f}\left(x\right)$ denote the first-order derivatives along the temporal and frequency directions, respectively. The gradient directly reflects the direction and magnitude of local intensity variations. However, a single gradient vector is sensitive to noise, slight misalignment, and isolated outliers, and is therefore not stable enough to represent neighborhood-level structural organization. For this reason, we do not use the gradient itself as the final local feature; instead, we further construct a second-order structure tensor.

The raw second-order tensor is defined as

(15) $$J_{0}\left(x\right)=\nabla L\left(x\right)\nabla L{\left(x\right)}^{T}=\left[\begin{array}{cc} L_{t}^{2}\left(x\right) & L_{t}\left(x\right)L_{f}\left(x\right)\\ L_{t}\left(x\right)L_{f}\left(x\right) & L_{f}^{2}\left(x\right) \end{array}\right].$$

This tensor encodes local directional energy, directional coupling, and anisotropy into a 2×2 symmetric matrix [7,8,9,32]. To lift this representation from a pointwise gradient outer product to a stable neighborhood-scale second-order structural statistic, we further apply local smoothing to $J_{0}\left(x\right)$:

(16) $$J\left(x\right)=G_{\sigma_{T}}*J_{0}\left(x\right),$$

where $G_{\sigma_{T}}$ is the Gaussian kernel corresponding to the integration scale of the structure tensor, and ∗ denotes convolution. This smoothing step is not merely denoising; rather, it accumulates directional information over a local neighborhood, so that the structure tensor more stably reflects the directional organization pattern inside a patch.

Based on this smoothed tensor, we add a ridge regularization term and define the pointwise structure tensor as

(17) $$S\left(x\right)=J\left(x\right)+\lambda I_{2},\;\lambda >0.$$

Thus, for any x∈Ω,

(18) $$S\left(x\right)\in S_{++}^{2}.$$

The membership in $S_{++}^{2}$ is ensured by two factors: local smoothing lifts the structural statistic from a pointwise gradient outer product to neighborhood-level second-order information, while $\lambda I_{2}$ further moves the matrix away from the degenerate boundary. This guarantees that subsequent Riemannian distances, Karcher means, and sample-level geometric comparisons are all performed in a valid SPD space.

Therefore, the entire patch can be represented as an SPD-valued structure field defined on Ω:

(19) S={S(x):x∈Ω}.

This representation is not a lossless transform of the raw patch, but a task-oriented local structural representation designed to preserve detection-relevant second-order geometric information, including directional organization, anisotropy, and spatial organization. All subsequent modeling and comparison steps are built around this SPD structure field.

### 2.3. Class-Conditional Reference Structure Fields Under AIRM

After obtaining the local SPD structure-field representation, the core training-stage task is to estimate, from the two classes of training samples, class-conditional reference structure fields that represent their local geometric organization patterns. Because the subsequent local comparison uses the affine-invariant Riemannian metric (AIRM), the reference centers must be defined under the same geometry. Otherwise, reference modeling and distance comparison would no longer share a unified intrinsic geometric framework.

Let the structure field of the *i*-th training patch in class c∈{0,1} be denoted by

(20) $$S_{i}^{\left(c\right)}=\left\{S_{i}^{\left(c\right)}\left(x\right):x\in \Omega \right\},\;S_{i}^{\left(c\right)}\left(x\right)\in S_{++}^{2}.$$

For any fixed position x∈Ω, the training samples from the same class provide a set of SPD matrices,

(21) $${\left\{S_{i}^{\left(c\right)}\left(x\right)\right\}}_{i=1}^{N_{c}},$$

where $N_{c}$ is the number of training samples in class *c*. Instead of globally compressing the entire patch first, this paper estimates the class-conditional reference structure tensor separately at each fixed position, thereby preserving the spatial distribution of local geometric relationships. The reference structure field for class *c* is therefore defined as

(22) $$S_{c}=\left\{{\bar{S}}_{c}\left(x\right):x\in \Omega \right\},\;c\in \left\{0,1\right\},$$

where ${\bar{S}}_{c}\left(x\right)$ is the class-conditional reference structure tensor at position *x*. For any $A,B\in S_{++}^{2}$, the AIRM geodesic distance is defined as [11,12,13]

(23) $$d_{R}\left(A,B\right)={∥\log\left(A^{-1/2}BA^{-1/2}\right)∥}_{F},$$

where log(·) denotes the matrix logarithm and ${∥\cdot ∥}_{F}$ denotes the Frobenius norm. This distance is affine-invariant on the SPD manifold and characterizes intrinsic geometric differences between covariance-like objects. It has therefore been widely used for geometric modeling and classification of SPD matrices.

Accordingly, the reference structure tensor of class *c* at position *x* is defined as the pointwise Karcher mean under AIRM [10,11,33]:

(24) $${\bar{S}}_{c}\left(x\right)=\arg\min_{Y\in S_{++}^{2}}\sum_{i=1}^{N_{c}}d_{R}^{2}\left(Y,S_{i}^{\left(c\right)}\left(x\right)\right).$$

The two class-conditional reference structure fields are thus given by

(25) $$S_{0}=\left\{{\bar{S}}_{0}\left(x\right):x\in \Omega \right\},\;S_{1}=\left\{{\bar{S}}_{1}\left(x\right):x\in \Omega \right\}.$$

This pointwise estimation strategy preserves the spatial distribution of class-conditional local structures and keeps the reference fields structurally aligned with the test structure field. Compared with the arithmetic mean, the Karcher mean is more consistent with the intrinsic geometry of the SPD manifold [14,24,34], and it also keeps reference estimation consistent with the subsequent AIRM-based distance comparison.

### 2.4. Local Distance-Difference Evidence

After obtaining the two class-conditional reference structure fields $S_{0}$ and $S_{1}$, the testing-stage task is to construct local distance-difference evidence by comparing the test structure field with the two reference fields under a unified SPD/AIRM geometry. Let the structure field corresponding to a test sample be

(26) $$S=\left\{S\left(x\right):x\in \Omega \right\},\;S\left(x\right)\in S_{++}^{2}.$$

Because the local structure tensor at each position belongs to the SPD space, the local comparison between the test structure field and the reference structure fields should not be reduced to element-wise Euclidean differences or Frobenius differences. Instead, it should be built on the intrinsic geometry that is consistent with reference modeling [13,14,15,16]. For this reason, AIRM is again adopted as the distance metric for pointwise comparison.

For any position x∈Ω, the pointwise geodesic distance from the test structure tensor to each class reference structure tensor is defined as

(27) $$d_{c}\left(x\right)=d_{R}\left(S\left(x\right),{\bar{S}}_{c}\left(x\right)\right),\;c\in \left\{0,1\right\}.$$

Here, ${\bar{S}}_{c}\left(x\right)$ is the pointwise Karcher mean reference tensor of class *c* at position *x*. The local distance-difference evidence is then defined pointwise as

(28) $$\Delta \left(x\right)=d_{0}^{2}\left(x\right)-d_{1}^{2}\left(x\right),\;x\in \Omega .$$

The sign of this quantity has a direct local discriminative meaning. When Δ(x)>0, the test structure tensor at position *x* is locally closer, in the geometric sense, to ${\bar{S}}_{1}\left(x\right)$ than to ${\bar{S}}_{0}\left(x\right)$. When Δ(x)<0, it is closer to ${\bar{S}}_{0}\left(x\right)$. Thus, Δ(x) forms a spatially distributed local distance-difference evidence map over the entire region Ω.

The key point of this construction is that local comparison and class-conditional reference modeling share the same geometric framework. In Section 2.3, the class-conditional centers are given by pointwise Karcher means under AIRM; in this section, the local comparison of the test sample with the reference centers is also performed using AIRM. Therefore, Δ(x) is not a comparison of raw intensity, gradient magnitude, or a single local energy value. It is a comparison of the relative closeness of local second-order structural objects on the SPD manifold. Accordingly, Δ(x) can be viewed as the pointwise expansion of the field-level relative-closeness statistic introduced in Section 2.1, characterizing the local geometric tendency of the test structure field toward the two reference fields.

Under this definition, Δ(x) is not merely an empirical distance-difference quantity; it can also be given an explicit information-geometric interpretation under local approximation conditions [13,35].

**Proposition** **1.** *Information-geometric interpretation of local distance-difference evidence. Suppose that, for any position x∈Ω, the test structure tensor S(x) satisfies a locally isotropic Riemannian Gaussian approximation model in a neighborhood of the class-c reference tensor ${\bar{S}}_{c}\left(x\right)$ [35]* *:*

(29) $$p_{c}\left(S\left(x\right)\right)\propto \exp\left[-\frac{d_{R}^{2}\left(S\left(x\right),{\bar{S}}_{c}\left(x\right)\right)}{2\sigma^{2}}\right],\;c\in \left\{0,1\right\},$$

*where $d_{R}^{2}\left(\cdot ,\cdot \right)$ is the squared AIRM geodesic distance and $\sigma^{2}$ is a shared local perturbation scale for the two classes. If the correlation among local positions is approximately absorbed into a nonnegative spatial weight w(x)≥0 in the field-level statistic, then the weighted distance-difference statistic*

(30) $$\sum_{x\in \Omega }w\left(x\right)\left[d_{R}^{2}\left(S\left(x\right),{\bar{S}}_{0}\left(x\right)\right)-d_{R}^{2}\left(S\left(x\right),{\bar{S}}_{1}\left(x\right)\right)\right]$$

*differs from the approximate log-likelihood ratio only by a positive proportionality factor and an additive constant.*

**Proof** **of** **Proposition** **1.** Under the locally isotropic Riemannian Gaussian approximation above,

(31) $$\log p_{c}\left(S\left(x\right)\right)=-\frac{d_{R}^{2}\left(S\left(x\right),{\bar{S}}_{c}\left(x\right)\right)}{2\sigma^{2}}+C_{c}\left(x\right),\;c\in \left\{0,1\right\}.$$

When the two classes share the same local perturbation scale and the difference between normalization constants is absorbed into an additive constant, we have

(32) $$\log\frac{p_{1}\left(S\left(x\right)\right)}{p_{0}\left(S\left(x\right)\right)}=-\frac{d_{R}^{2}\left(S\left(x\right),{\bar{S}}_{1}\left(x\right)\right)}{2\sigma^{2}}+\frac{d_{R}^{2}\left(S\left(x\right),{\bar{S}}_{0}\left(x\right)\right)}{2\sigma^{2}}+C.$$

Rearranging gives

(33) $$\log\frac{p_{1}\left(S\left(x\right)\right)}{p_{0}\left(S\left(x\right)\right)}=\frac{1}{2\sigma^{2}}\left[d_{R}^{2}\left(S\left(x\right),{\bar{S}}_{0}\left(x\right)\right)-d_{R}^{2}\left(S\left(x\right),{\bar{S}}_{1}\left(x\right)\right)\right]+C,$$

that is,

(34) $$\log\frac{p_{1}\left(S\left(x\right)\right)}{p_{0}\left(S\left(x\right)\right)}=\frac{1}{2\sigma^{2}}\Delta \left(x\right)+C.$$

Further taking a spatially weighted sum over x∈Ω yields

(35) $$\sum_{x\in \Omega }w\left(x\right)\log\frac{p_{1}\left(S\left(x\right)\right)}{p_{0}\left(S\left(x\right)\right)}=\frac{1}{2\sigma^{2}}\sum_{x\in \Omega }w\left(x\right)\Delta \left(x\right)+C^{\prime }.$$

Therefore, under the local approximation conditions above, the field-level weighted distance-difference statistic is monotonically consistent with the approximate log-likelihood ratio. This proposition explains only the information-geometric meaning of the additive distance-difference statistic under a locally isotropic Riemannian Gaussian approximation. The discriminative weighting and block-wise robust pooling introduced below are sample-level aggregation mechanisms built on this local distance-difference evidence. They are intended to enhance stable discriminative regions and reduce the effects of local misalignment, structural fragmentation, and abnormal responses, but they do not constitute an optimality proof in the Neyman–Pearson sense. □

### 2.5. Discriminative Weighting and Block-Wise Robust Pooling

The local distance-difference evidence map Δ(x) provides spatially distributed geometric information, but different positions do not contribute equally to the final decision. To enhance stable discriminative regions and suppress the effects of local misalignment, structural fragmentation, and abnormal responses, we introduce discriminative weighting and block-wise robust evidence pooling.

Let ${\bar{S}}_{0}\left(x\right)$ and ${\bar{S}}_{1}\left(x\right)$ denote the two class-conditional reference structure tensors at position *x*. The local between-class separation is defined as

(36) $$\mathrm{Sep}\left(x\right)=d_{R}^{2}\left({\bar{S}}_{0}\left(x\right),{\bar{S}}_{1}\left(x\right)\right).$$

For class c∈{0,1}, the within-class dispersion at position *x* is defined as

(37) $${\mathrm{Disp}}_{c}\left(x\right)=\frac{1}{N_{c}}\sum_{i=1}^{N_{c}}d_{R}^{2}\left(S_{i}^{\left(c\right)}\left(x\right),{\bar{S}}_{c}\left(x\right)\right).$$

The unnormalized discriminative weight is then given by

(38) $$W_{\mathrm{raw}}\left(x\right)=\frac{\mathrm{Sep}(x)}{{\mathrm{Disp}}_{0}\left(x\right)+{\mathrm{Disp}}_{1}\left(x\right)+\varepsilon_{w}},\;\varepsilon_{w}>0.$$

This quantity measures the stable local separability of the geometric structure at position *x*. Positions with large between-class separation and small within-class dispersion are expected to provide persistent discriminative evidence, whereas positions dominated by high dispersion and low separation are down-weighted.

To make the weights comparable across spatial positions and reduce high-frequency fluctuations caused by finite training samples, $W_{\mathrm{raw}}\left(x\right)$ is first min–max normalized,

(39) $$W_{0}\left(x\right)=\mathrm{MinMax}\left(W_{\mathrm{raw}}\left(x\right)\right),$$

and then mildly smoothed and clipped to the interval [0,1]:

(40) $$W\left(x\right)=\mathrm{clip}\left(G_{\sigma_{W}}*W_{0}\left(x\right),0,1\right).$$

The resulting discriminative weight map W(x) emphasizes stable local discriminative regions while preserving spatial continuity in the weight field.

After obtaining Δ(x) and W(x), the remaining task is to aggregate the weighted local distance-difference evidence into a sample-level detection statistic. A direct global summation of the weighted evidence,

$$\sum_{x\in \Omega }W\left(x\right)\Delta \left(x\right),$$

can still be sensitive to local shifts, missing structures, and isolated abnormal responses. In complex non-stationary scenarios, discriminative evidence is often concentrated in a small number of local structural regions rather than uniformly distributed over the entire patch. This idea is related to multiple-instance learning and set-level pooling, where sample-level decisions are inferred from local instances. However, the proposed method uses non-learned, geometry-driven evidence aggregation rather than trainable attention weights or deep pooling functions [36,37,38].

We define a set of overlapping blocks over Ω:

(41) $$B={\left\{B_{\ell }\right\}}_{\ell =1}^{L}.$$

For each block $B_{\ell }$, the weighted block score is computed as follows:

(42) $$s_{\ell }=\frac{\sum_{x\in B_{\ell }}W\left(x\right)\Delta \left(x\right)}{\sum_{x\in B_{\ell }}W\left(x\right)+\varepsilon_{b}},\;\ell =1,\;\ldots ,\;L,\;\varepsilon_{b}>0.$$

This block-level averaging reduces the influence of pointwise outliers and small local shifts, while preserving the spatial concentration of high-response local structures.

The block scores ${\left\{s_{\ell }\right\}}_{\ell =1}^{L}$ are then sorted in descending order. Given a top-block fraction q∈(0,1], the number of selected high-response blocks is

(43) K=max{1,⌊qL⌋}.

The global mean term and the top-block mean term are defined as

(44) $$\mu_{\mathrm{all}}=\frac{1}{L}\sum_{\ell =1}^{L}s_{\ell },$$

(45) $$\mu_{\mathrm{top}}=\frac{1}{K}\sum_{\ell \in L_{\mathrm{top}}}s_{\ell },$$

where $L_{\mathrm{top}}$ denotes the index set of the selected top *K* blocks. The final sample-level statistic is

(46) $$T=\left(1-\alpha \right)\mu_{\mathrm{all}}+\alpha \mu_{\mathrm{top}},\;\alpha \in \left[0,1\right].$$

Here, $\mu_{\mathrm{all}}$ captures the overall geometric tendency over the field, whereas $\mu_{\mathrm{top}}$ emphasizes salient responses from sparse discriminative structures [39]. The aggregation coefficient α controls the trade-off between global stability and local saliency. This aggregation is not intended as a theoretically optimal mixture; rather, it provides a controlled balance for local-structure detection, where discriminative evidence can be spatially sparse and locally unstable. In the experiments, the top-block fraction *q* and the aggregation coefficient α are fixed across all nominal signal-to-noise ratio/signal-to-clutter ratio (SNR/SCR)-like index points, random seeds, and proposed and ablated variants; they are not re-tuned separately for individual operating points. The general selection principles of these and other numerical parameters are summarized in the subsection entitled “Controlled SPD-Field Benchmark Generation”.

Thus, the test sample is mapped from the local distance-difference evidence map Δ(x) to a sample-level statistic *T*. The discriminative weight map emphasizes stable discriminative positions, block-wise scoring reduces pointwise anomalies and local shifts, and the combination of $\mu_{\mathrm{all}}$ and $\mu_{\mathrm{top}}$ balances global stability with local saliency.

### 2.6. Final Decision Rule and Fixed-$P_{\mathrm{Fa}}$ Threshold Calibration

Given the sample-level statistic *T*, the final decision rule is defined as

(47) $$T\overset{H_{1}}{\underset{H_{0}}{≷}}\gamma .$$

Here, γ is the detection threshold. A larger value of *T* indicates that the test sample is closer to the target-class reference structure field $S_{1}$ after discriminative weighting and robust pooling; conversely, a smaller value indicates greater closeness to the null-class reference structure field $S_{0}$. Therefore, the threshold is calibrated using an independent $H_{0}$ calibration set [40,41,42].

Let the statistics computed from the independent $H_{0}$ calibration set be ${\left\{T_{j}^{\mathrm{cal},0}\right\}}_{j=1}^{M_{\mathrm{cal}}}$. Sorting them in ascending order gives

$$T_{\left(1\right)}^{\mathrm{cal},0}\leq T_{\left(2\right)}^{\mathrm{cal},0}\leq \;\cdots \;\leq T_{\left(M_{\mathrm{cal}}\right)}^{\mathrm{cal},0}.$$

For a target false-alarm probability $P_{\mathrm{fa}}$, we use the empirical order-statistic threshold

(48) $$k\left(P_{\mathrm{fa}}\right)=\min\left\{\max\left[\left\lceil \left(1-P_{\mathrm{fa}}\right)M_{\mathrm{cal}}\right\rceil ,1\right],M_{\mathrm{cal}}\right\},\;\gamma \left(P_{\mathrm{fa}}\right)=T_{\left(k\left(P_{\mathrm{fa}}\right)\right)}^{\mathrm{cal},0}.$$

The final decision is

(49) $$T>\gamma \left(P_{\mathrm{fa}}\right)\Rightarrow H_{1},\;T\leq \gamma \left(P_{\mathrm{fa}}\right)\Rightarrow H_{0}.$$

Because $\gamma (P_{\mathrm{fa}})$ is estimated from a finite number of $H_{0}$ calibration samples, the achieved false-alarm probability does not necessarily equal the nominal target value exactly. We therefore estimate empirical false-alarm probability, referred to as achieved $P_{\mathrm{fa}}$, using an independent $H_{0}$ audit set:

(50) $${\hat{P}}_{\mathrm{fa}}^{\mathrm{audit}}=\frac{1}{M_{\mathrm{audit}}}\sum_{j=1}^{M_{\mathrm{audit}}}1\left(T_{j}^{\mathrm{audit},0}>\gamma \left(P_{\mathrm{fa}}\right)\right).$$

The detection probability is estimated using an independent $H_{1}$ test set:

(51) $${\hat{P}}_{d}=\frac{1}{M_{1}}\sum_{j=1}^{M_{1}}1\left(T_{j}^{\mathrm{test},1}>\gamma \left(P_{\mathrm{fa}}\right)\right).$$

This protocol explicitly separates threshold estimation, detection evaluation, and false-alarm auditing. It avoids calibration/test leakage and ensures that fixed-$P_{\mathrm{fa}}$ comparisons among different detectors are performed on a consistent experimental basis. Receiver operating characteristic (ROC) curves are obtained by sweeping the threshold, whereas all fixed-$P_{\mathrm{fa}}$ operating-point results use the independent $H_{0}$ calibration set for threshold determination and the independent $H_{0}$ audit set for false-alarm validation.

### 2.7. Algorithmic Summary of the Proposed Detector

**Figure 1:**
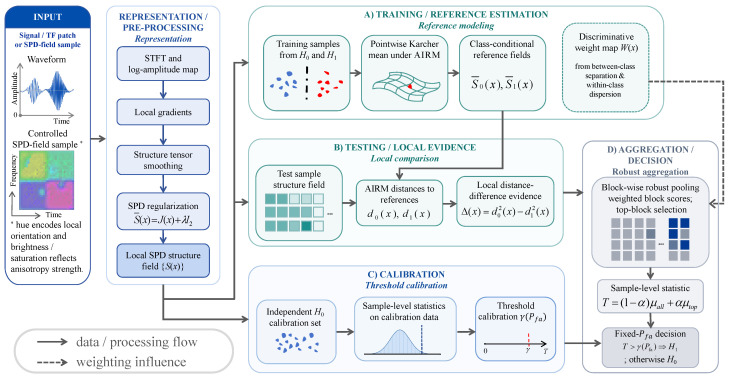
Method overview of the proposed local SPD structure-field detector. Solid arrows denote data or processing flow, whereas dashed arrows indicate the influence of the discriminative weight map on the aggregation stage. For raw waveform or time–frequency inputs, the local SPD structure field is constructed through STFT/log-amplitude representation, local gradients, structure-tensor smoothing, and SPD regularization. In the controlled SPD-field benchmark, generated SPD-field samples enter the reference-estimation, calibration, and testing stages directly. The detector then estimates pointwise Karcher mean reference fields, constructs local distance-difference evidence, performs block-wise robust aggregation, and makes a fixed-$P_{\mathrm{fa}}$ decision using an independently calibrated threshold. Abbreviations in the figure: TF, time–frequency; SPD, symmetric positive definite; STFT, short-time Fourier transform; AIRM, affine-invariant Riemannian metric; H0, null hypothesis; H1, alternative hypothesis; Pfa, false-alarm probability.

The leading idea of the proposed detector is to compare local structural organization rather than raw intensity or a single global statistic. As illustrated in Figure 1, a waveform or time–frequency patch is first represented as a local SPD structure field. Class-conditional reference fields are then estimated by pointwise Karcher means under AIRM. During testing, the test field is compared with the two reference fields to obtain local distance-difference evidence, which is weighted, robustly pooled, and finally converted into a fixed-$P_{\mathrm{fa}}$ decision using an independently calibrated threshold.

Algorithm 1 summarizes the complete procedure. For raw waveform or spectrogram inputs, the local SPD structure field is constructed using the STFT and structure-tensor procedure described in Section 2.2. In the controlled benchmark of Section 3, generated SPD fields are directly used as inputs to the reference-estimation and scoring stages.


**Algorithm 1.** Proposed information-geometric detector based on local SPD structure fields
**Input:**
Raw training samples or precomputed SPD-field samples $X_{i}^{\left(0\right)}$, $X_{i}^{\left(1\right)}$/$S_{i}^{\left(0\right)}$, $S_{i}^{\left(1\right)}$;raw test sample or precomputed SPD-field sample X/S;independent $H_{0}$ calibration set ${\left\{X_{j}^{\mathrm{cal},0}\right\}}_{j=1}^{M_{\mathrm{cal}}}$;target false-alarm probability $P_{\mathrm{fa}}$;local patch support Ω;overlapping block set $B={\left\{B_{\ell }\right\}}_{\ell =1}^{L}$;top-block fraction *q*;aggregation coefficient α.
**Output:**
Sample-level detection statistic *T* and final decision.
**Training phase:**

1:For each training sample, obtain its local SPD structure field.2:If raw inputs are used, construct the local SPD structure field following Section 2.2.3:If the input is a controlled SPD-field sample, use the generated SPD field directly.4:End for.5:For each position x∈Ω and each class c∈{0,1}, estimate the pointwise Karcher mean reference tensor according to Equation (24).6:Obtain the two class-conditional reference fields $S_{0}$ and $S_{1}$.7:Compute Sep(x), ${\mathrm{Disp}}_{0}\left(x\right)$, and ${\mathrm{Disp}}_{1}\left(x\right)$ for all x∈Ω.8:Construct the discriminative weight map W(x) by normalization, smoothing, and clipping.

**Calibration phase:**

9:For each calibration sample $X_{j}^{\mathrm{cal},0}$, do.10:Compute its local SPD structure field $S_{j}^{\mathrm{cal},0}$.11:Compute the local distance-difference evidence Δ(x) according to Equation (28).12:For each block $B_{\ell }$, compute the weighted block score $s_{\ell }$ according to Equation (42).13:Sort ${\left\{s_{\ell }\right\}}_{\ell =1}^{L}$ in descending order and select the top K=max{1,⌊qL⌋} blocks.14:Compute the calibration statistic *T* according to Equation (46).15:End for.16:Sort the calibration $H_{0}$ statistics and obtain $\gamma (P_{\mathrm{fa}})$ using the empirical order-statistic rule in Section 2.6.

**Testing phase:**

17:Compute the local SPD structure field S of the test sample *X*.18:Compute the local distance-difference evidence Δ(x) according to Equation (28).19:For each block $B_{\ell }$, compute the weighted block score $s_{\ell }$ according to Equation (42).20:Sort ${\left\{s_{\ell }\right\}}_{\ell =1}^{L}$ in descending order and select the top *K* blocks.21:Compute $\mu_{\mathrm{all}}$ and $\mu_{\mathrm{top}}$ according to Equations (44) and (45).22:Compute the final statistic $T=\left(1-\alpha \right)\mu_{\mathrm{all}}+\alpha \mu_{\mathrm{top}}$.23:If $T>\gamma (P_{\mathrm{fa}})$, decide $H_{1}$; otherwise, decide $H_{0}$.



### 2.8. Computational Complexity

Table 1 summarizes the computational complexity of the proposed detector. Let $N=N_{0}+N_{1}$ denote the total number of training samples, |Ω| the number of spatial positions, $I_{K}$ the maximum number of Karcher iterations, *L* the number of overlapping blocks, $|B_{\ell }|$ the size of block $B_{\ell }$, *M* the number of calibration samples, and $C_{\det}$ the cost of evaluating one sample-level statistic.

The main computational cost of the proposed detector comes from pointwise Riemannian reference estimation and repeated AIRM distance evaluation. Although AIRM provides an intrinsic comparison on the SPD manifold, faster alternatives such as Log-Euclidean metrics or tangent-space approximations may reduce the online cost in large-scale settings [24,34,43].

## 3. Experimental Results

### 3.1. Experimental Setup and Main Detection Scene

The experiments evaluate the effectiveness, component contributions, and applicability limits of the proposed detector. In all formal experiments, the training, calibration, testing, and audit sets are mutually independent. Receiver operating characteristic (ROC) curves are obtained by directly sweeping the detection threshold. For fixed false-alarm probability operating points, the threshold is determined from the empirical quantile of an independent $H_{0}$ calibration set matched to the corresponding experimental condition. Two target operating points, $P_{\mathrm{fa}}=10^{-2}$ and $P_{\mathrm{fa}}=10^{-3}$, are reported throughout the experiments. The achieved false-alarm probability is also reported as an empirical calibration check to verify whether the nominal false-alarm target is realized at the expected order of magnitude.

The main experiment uses a structured-locality detection scene. This scene is not designed to create separability through pronounced global energy differences, mean spectral appearance differences, or a single pooled covariance statistic. Instead, the class difference is mainly encoded in local structural orientation, spatial arrangement, and structural stability. From the viewpoint of non-stationary signal analysis, this scene represents the situation in which the informative content is spatially inhomogeneous and locally organized over the time–frequency support. The local structural pattern varies with position, and the class distinction is expressed through localized orientation organization and spatial arrangement rather than through a single global distribution. Therefore, the benchmark is intended to emulate a local-structure-dominated non-stationary detection setting at the SPD-field object layer. Additional structural perturbation and non-Gaussian background experiments are further conducted to examine the robustness and applicability limits of the proposed detector.

In the formal experiments, 20,000 independent $H_{0}$ samples are used for fixed-$P_{\mathrm{fa}}$ threshold calibration, 500 $H_{0}$ test samples and 500 $H_{1}$ test samples are used for ROC, area under the curve (AUC), and detection probability ($P_{d}$) evaluation, and another 20,000 independent $H_{0}$ audit samples are used only for achieved $P_{\mathrm{fa}}$ validation. All evaluated methods share the same data splits, target false-alarm probabilities, calibration protocol, and audit protocol. Therefore, the performance differences are mainly attributable to the detection statistics themselves rather than to threshold implementation or sample reuse.

The experiments are conducted on a controlled SPD-field object-layer benchmark. Section 2 describes the complete construction, from raw observations or time–frequency patches to local SPD structure fields. In Section 3, however, samples are generated directly at the SPD-field object layer in order to isolate and evaluate the core detector mechanisms after the representation stage, including reference-field estimation, local distance-difference evidence, discriminative weighting, block-wise robust pooling, and fixed-$P_{\mathrm{fa}}$ calibration. This design allows the experiment to focus on whether the proposed detector can exploit local structural organization once such structures have been represented as SPD fields. Therefore, the experimental conclusions validate the detector mechanism after SPD-field representation, rather than a complete end-to-end waveform-level detection pipeline.

#### Controlled SPD-Field Benchmark Generation

The main scene generates samples directly at the local SPD structure-field object layer. The patch support is defined as

(52) Ω={1, …, H}×{1, …, W},H=W=32.

Each sample is represented as an SPD-valued field,

(53) $$S_{i}=\left\{S_{i}\left(x\right):x\in \Omega \right\},\;S_{i}\left(x\right)\in S_{++}^{2}.$$

For an orientation angle θ, the directional SPD prototype matrix is defined as

(54) $$C\left(\theta \right)=R\left(\theta \right)\left[\begin{array}{cc} \lambda_{1} & 0\\ 0 & \lambda_{2} \end{array}\right]R{\left(\theta \right)}^{T},$$

where R(θ) is a two-dimensional rotation matrix, $\lambda_{1}=1.80$, and $\lambda_{2}=0.55$. All generated matrices are symmetrized and constrained by a minimum eigenvalue condition,

(55) $$\lambda_{\min}\left(S_{i}\left(x\right)\right)\geq 10^{-4}.$$

The background structure is generated from a shared orientation atlas $\theta_{\mathrm{bg}}^{\mathrm{atlas}}\left(x\right)$. This atlas is constructed from four quadrant-wise base orientations, −55°, −15°, 35°, and 70°, and is mildly smoothed by a Gaussian kernel to avoid artificial discontinuities. At background positions, the SPD tensor is generated in the log domain as

(56) $$S_{\mathrm{bg}}\left(x\right)=\Pi_{S_{++}^{2}}\left[\exp\left(\log C\left(\theta_{\mathrm{bg}}^{\mathrm{atlas}}\left(x\right)+\varepsilon_{\theta }\right)+E_{\mathrm{bg}}\right)\right],$$

where $\varepsilon_{\theta }$ denotes background orientation jitter, $E_{\mathrm{bg}}$ is a symmetric log-domain perturbation, and $\Pi_{S_{++}^{2}}$ denotes SPD correction under the eigenvalue-flooring constraint.

Class-discriminative differences are introduced through stable local structural islands. The class-defining orientation separation is set to $\theta_{\mathrm{sep}}=44{}^{\circ}$. With the base orientation set to 0°, the two class-discriminative orientations are

(57) $$\theta_{\mathrm{disc}}^{\left(0\right)}=-\frac{\theta_{\mathrm{sep}}}{2},\;\theta_{\mathrm{disc}}^{\left(1\right)}=+\frac{\theta_{\mathrm{sep}}}{2}.$$

The main scene contains three discriminative local structures centered at (8,8), (12,22), and (23,14), with a radius of 3.2 and relative amplitudes of 1.00, 0.82, and 0.68, respectively. The mask of the *k*-th local structure is defined as

(58) $$M_{k}\left(x\right)=a_{k}\frac{\exp\left(-\frac{∥x-c_{k}{∥}^{2}}{2r^{2}}\right)}{\max_{x\in \Omega }\exp\left(-\frac{∥x-c_{k}{∥}^{2}}{2r^{2}}\right)}.$$

Inside a discriminative local structure, the local orientation is obtained by interpolating between the background orientation and the class-discriminative orientation:

(59) $$\theta_{\mathrm{loc}}^{\left(c\right)}\left(x\right)=\arg\left[\left(1-M_{k}\left(x\right)\right)e^{j\theta_{\mathrm{bg}}\left(x\right)}+M_{k}\left(x\right)e^{j\theta_{\mathrm{disc}}^{\left(c\right)}}\right]+\varepsilon_{\mathrm{disc}}.$$

The discriminative orientation jitter is set to 0.4°, and the discriminative log-domain perturbation amplitude is set to 0.006.

To avoid reducing the scene to a simple template-matching problem, each sample further includes six shared unstable distractors. The center of each distractor is randomly sampled, its radius is set to 2.2, its orientation is randomly selected from

{−75°, −45°, −15°, 15°, 45°, 75°},

and a rotation jitter of 16° is applied. Its amplitude is randomly sampled from [0.22,0.48]. These distractors serve as nuisance local structures and simulate local interference, misalignment, and non-discriminative structural responses. Consequently, each generated sample is a spatially inhomogeneous SPD field rather than a homogeneous field generated from a single position-independent tensor distribution. The background atlas, localized discriminative islands, orientation jitter, and shared unstable distractors jointly create position-dependent local structural variations. These variations are used to represent, at the object layer, the kind of non-stationary time–frequency organization that motivates the proposed detector.

In this benchmark, the nominal index ρ is a background-difficulty control parameter rather than a measured physical signal-to-noise ratio or signal-to-clutter ratio. It does not modify the class-defining structural separation or the amplitudes of the discriminative local structures. Instead, ρ controls nuisance factors, including background perturbation, background orientation jitter, distractor perturbation, and distractor strength. Although the benchmark does not define a physical SCR, the nominal index serves an analogous role by controlling the strength of background nuisance factors.

The perturbation scale is defined as

(60) $$\eta \left(\rho \right)=\mathrm{clip}\left[10^{(\rho_{\mathrm{ref}}-\rho )/s},\eta_{\min},\eta_{\max}\right],$$

where $\rho_{\mathrm{ref}}=-12$ dB, s=20, $\eta_{\min}=0.75$, and $\eta_{\max}=2.50$ in the main experiments. The exponential form in Equation (60) is not intended to model a physical SNR or SCR relationship. It is adopted as a convenient monotonic mapping that produces approximately logarithmic changes in nuisance intensity when the nominal index is varied in dB. Therefore, the nominal SNR/SCR-like index sweep evaluates how the detectors behave under different background-difficulty conditions. It facilitates fixed-$P_{\mathrm{fa}}$ performance comparison across nuisance levels, while avoiding artificial separability caused by changing the class-defining local structures themselves.

All experiments use mutually independent training, calibration, testing, and audit sets. The training sets are used to estimate class-conditional pointwise reference fields and the discriminative weight map. The calibration set is used only for fixed-$P_{\mathrm{fa}}$ threshold estimation. The testing sets are used for AUC and $P_{d}$ evaluation, and the independent $H_{0}$ audit set is used only for achieved $P_{\mathrm{fa}}$ validation.

The parameters listed in Table 2 are not used as free fitting degrees of freedom for individual curves or operating points. They are fixed before the formal comparisons and kept unchanged across nominal SNR/SCR-like index values, random seeds, calibration/test splits, and proposed and ablated variants, unless an experiment explicitly studies a perturbation factor. This fixed-configuration policy is intended to avoid treating the detector as an unconstrained multi-parameter fitting model.

The selection of these parameters follows the principles of numerical stability, local-structure preservation, SPD validity, robust aggregation, and fair fixed-$P_{\mathrm{fa}}$ comparison. Specifically, $\epsilon_{\alpha }$ is used as a small stabilization term to prevent logarithmic singularities; $\sigma_{T}$ sets the local integration scale of the structure tensor; λ and the eigenvalue floor keep tensors inside the valid SPD cone; $\epsilon_{w}$ and $\sigma_{W}$ stabilize the discriminative weight map; the block size, stride, and $\epsilon_{b}$ control robust local pooling; *q* and α balance sparse local saliency with global stability; the Karcher tolerance and iteration limit are determined by convergence and computational cost; and the calibration, test, and audit sample sizes are used to separate threshold calibration, detection evaluation, and achieved-$P_{\mathrm{fa}}$ auditing. The selected values are not claimed to be universally optimal; rather, they provide a fixed and transparent configuration for comparing detector mechanisms under the same controlled benchmark and calibration protocol.

### 3.2. Main Results Under the Structured-Locality Detection Scene

This section compares the proposed detector with the uniform-weight variant and the global-pooling variant in the main scene. The nominal SNR/SCR-like background-difficulty index is swept from −20 dB to 0 dB. Both the overall ranking ability and the detection performance at fixed-$P_{\mathrm{fa}}$ operating points are evaluated under this controlled nuisance-difficulty sweep.

**Figure 2:**
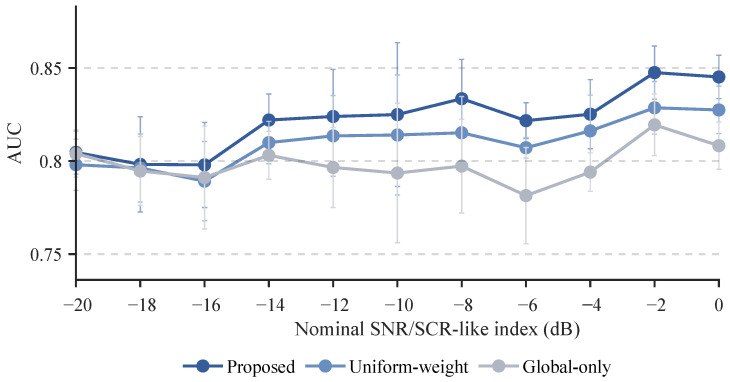
AUC versus the nominal SNR/SCR-like background-difficulty index under the main structured-locality detection scene. The index controls nuisance/background difficulty in the controlled SPD-field benchmark. As the nominal index decreases, the nuisance intensity increases, while the class-defining local structures remain unchanged.

As shown in Figure 2, the proposed detector achieves the highest average AUC in the main scene, but the source of this advantage should be interpreted in layers. Compared with the global-pooling variant, the proposed detector shows a more consistent advantage across the nominal SNR/SCR-like index sweep, indicating that purely global aggregation weakens the contribution of local discriminative structures. Compared with the uniform-weight variant, the improvement is smaller but generally stable, suggesting that discriminative weighting provides a supplementary benefit by emphasizing stable local structural regions. Because the benchmark deliberately suppresses global low-order cues, the AUC does not vary strictly monotonically with the nominal SNR/SCR-like index. Local fluctuations between adjacent nominal SNR/SCR-like index points mainly reflect the joint effects of nuisance/background variability, random distractors, and the finite number of random seeds, rather than a simple energy-detection behavior.

**Figure 3:**
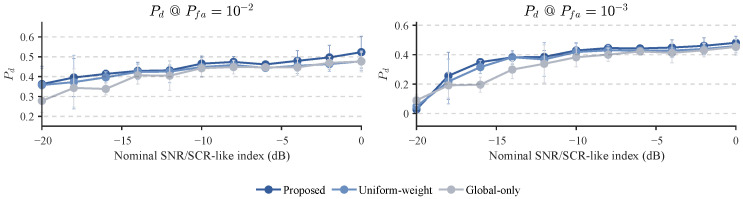
Detection probability curves at fixed-$P_{\mathrm{fa}}$ operating points versus the nominal SNR/SCR-like background-difficulty index under the main structured-locality detection scene. The index controls background perturbation, orientation jitter, distractor perturbation, and distractor strength, and is used here to facilitate performance comparison under different controlled nuisance levels. As the nominal index decreases, the nuisance intensity increases while the class-defining local structures remain unchanged.

Figure 3 presents the fixed-$P_{\mathrm{fa}}$ detection probability curves under different values of the nominal SNR/SCR-like background-difficulty index. These curves are intended to facilitate performance comparison under controlled nuisance levels, in the same spirit as comparing detection probability under different signal-to-noise or signal-to-clutter conditions. Although the benchmark does not define a physical signal-to-clutter ratio, the nominal index plays an analogous role by controlling the intensity of background nuisance factors while keeping the class-defining local structures unchanged.

At fixed-$P_{\mathrm{fa}}$ operating points, the proposed detector performs better overall than the uniform-weight and global-pooling variants. At $P_{\mathrm{fa}}=10^{-2}$, the detection probabilities at −20 dB, −12 dB, and 0 dB are 0.3624, 0.4316, and 0.5232, respectively, all of which are higher than those of the two simplified variants. At $P_{\mathrm{fa}}=10^{-3}$, the advantage of the proposed detector is generally retained, and it achieves the highest or joint-highest performance in the mid-to-high nominal SNR/SCR-like index range. This fixed-$P_{\mathrm{fa}}$ advantage should be interpreted together with the paired-difference analysis in Section 3.3, because the gains over geometry-simplified structure-field baselines are clearly smaller than the gains over global low-order baselines.

### 3.3. Baseline Comparison and Ablation Analysis

**Figure 4:**
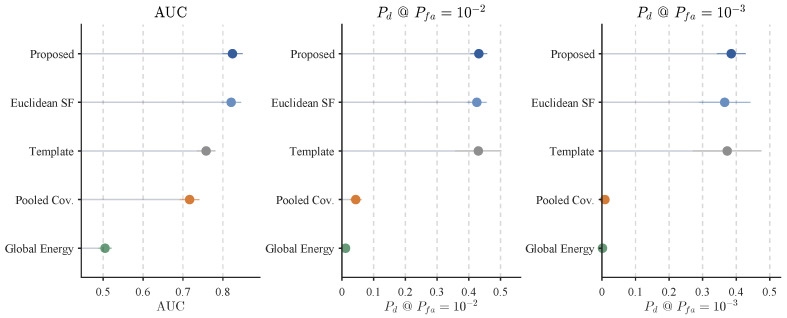
Baseline comparison under the main scene.

To assess comparability against external baselines and the contribution of individual components, we further conduct baseline comparisons and ablation analyses in the main scene. All methods use the same data splits and the same fixed-$P_{\mathrm{fa}}$ calibration protocol. Therefore, the comparison focuses on the choice of detection object and the construction of the sample-level statistic, rather than on differences in threshold implementation. Figure 4 summarizes the baseline comparison under the main scene, and Table 3 reports the corresponding average performance results.

The baseline comparison shows that local structure-field methods clearly outperform the global-energy and pooled-covariance baselines. This indicates that, in the present main scene, the discriminative information mainly resides in local structural organization rather than in global energy or a single pooled covariance statistic. The template-correlation baseline is competitive at fixed-$P_{\mathrm{fa}}$ operating points, but its AUC is substantially lower than those of local structure-field methods, suggesting that simple template similarity cannot fully capture the geometric variations in local SPD structure fields. The achieved $P_{\mathrm{fa}}$ values of all methods are close to their corresponding target levels, supporting a fair comparison under fixed-$P_{\mathrm{fa}}$ conditions.

Within the structure-field baselines, the proposed detector provides only a modest improvement over the Euclidean/Frobenius structure-field baseline. To avoid over-interpreting small mean differences, we further conduct paired-difference analysis. For any metric

$$m\in \{\mathrm{AUC},P_{d}@10^{-2},P_{d}@10^{-3}\},$$

given a baseline *b* and a paired experimental unit *u*, the paired difference is defined as

(61) $$\Delta m_{u,b}=m_{u,\mathrm{proposed}}-m_{u,b}.$$

For the main nominal SNR/SCR-like index sweep, the paired difference is first computed for each seed–index pair and then averaged over all nominal SNR/SCR-like index points within each random seed. Therefore, the independent paired unit for the confidence interval is the random seed rather than an individual seed–index pair. This prevents adjacent nominal SNR/SCR-like index points under the same seed from being treated as fully independent observations and reduces the risk of underestimating the confidence interval.

We report the mean paired difference and its 95% confidence interval. This analysis is used to avoid over-interpreting small mean performance differences.

(62) $${\bar{\Delta m}}_{b}\pm t_{0.975,n-1}\frac{s_{\Delta m,b}}{\sqrt{n}}.$$

Table 4 reports the resulting paired performance differences between the proposed detector and the competing methods.

**Figure 5:**
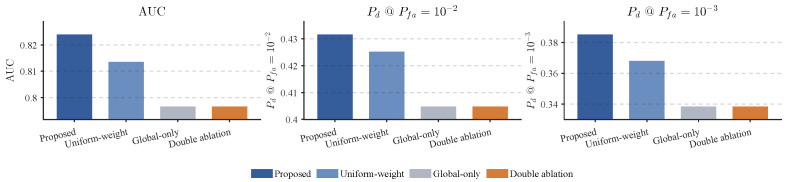
Ablation analysis under the main scene.

The seed-averaged paired-difference analysis shows that the proposed detector achieves positive AUC gains over both the uniform-weight and global-pooling variants on the main sweep. Its fixed-$P_{\mathrm{fa}}$ gains over the global-pooling variant are also relatively clear. For the uniform-weight variant, the confidence interval of the $P_{d}$ difference at $P_{\mathrm{fa}}=10^{-3}$ slightly crosses zero after seed-level aggregation; therefore, the improvement at this operating point should be interpreted cautiously. In contrast, the gains over global low-order baselines are much larger, whereas the gains over the Euclidean/Frobenius structure-field baseline are small. The main evidence therefore supports the effectiveness of the local SPD structure-field object layer. AIRM-based geometry-consistent modeling should be interpreted as a smaller supplementary contribution rather than as the sole source of a large performance jump. Figure 5 presents the ablation analysis under the main scene.

In the ablation analysis, the proposed detector, the uniform-weight variant, and the global-pooling variant share the same local SPD structure-field representation and the same pointwise AIRM distance-difference map. They differ only in whether discriminative weighting and top-block emphasis are used. The results indicate that the proposed detector achieves the best values on all three core metrics. This suggests that useful discriminative information is not uniformly distributed over the whole patch, but is concentrated in several stable local structural regions. Discriminative weighting increases the contribution of these regions, while block-wise pooling reduces the influence of low-information areas and local abnormal responses on the sample-level statistic.

### 3.4. Robustness Under Structural Perturbations and Non-Gaussian Conditions

**Figure 6:**
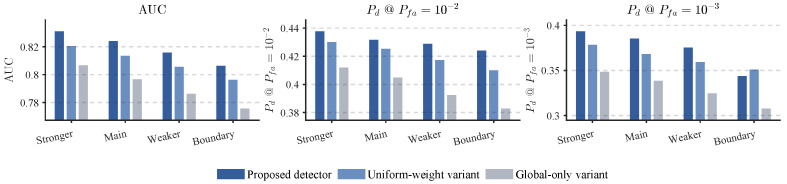
Robustness under local structural perturbations.

This section evaluates robustness under local structural perturbations and non-Gaussian background perturbations. The purpose is to examine whether the performance ordering remains stable when structural strength and background distribution change. Figure 6 reports the robustness results under local structural perturbations.

In the local structural perturbation experiment, the structural-strength perturbation results show that the gain of the proposed detector over the uniform-weight variant remains generally stable across perturbation settings around the main scene. Under the slightly stronger, main, slightly weaker, and boundary auxiliary conditions, ΔAUC remains positive and stays on the order of $10^{-2}$. This indicates that, when the orientation separation and stability of local structures undergo small to moderate changes, the ranking advantage of the proposed detector does not reverse. The result suggests that the proposed detector retains its advantage when local structures remain present but their strength and stability are perturbed.

The non-Gaussian background perturbation experiment compares Gaussian, Laplace-distributed, impulsive, and heavy-tailed field-level background perturbations. Perturbations are applied at the SPD-field object layer. For an SPD tensor S(x) at position *x*, the tensor is first symmetrized, and a symmetric random perturbation is then added in the log domain:

(63) $$\tilde{S}\left(x\right)=\Pi_{S_{++}^{2}}\left[\exp\left(\log\left(\frac{S\left(x\right)+S{\left(x\right)}^{T}}{2}\right)+\Delta_{\xi }\left(x\right)\right)\right],$$

where $\Delta_{\xi }\left(x\right)=\Delta_{\xi }{\left(x\right)}^{T}$ is a symmetric random perturbation matrix, and $\Pi_{S_{++}^{2}}$ denotes SPD correction under the same minimum eigenvalue constraint used in the main scene, namely $\lambda_{\min}\geq 10^{-4}$. The perturbation matrix is defined as

(64) $$\Delta_{\xi }\left(x\right)=\sigma_{\xi }\frac{Z_{\xi }\left(x\right)+Z_{\xi }{\left(x\right)}^{T}}{2},$$

where $\sigma_{\xi }$ is the noise scale.

Four field-level perturbations are compared. In Gaussian perturbation, the entries of $Z_{\xi }$ are independently sampled from the standard normal distribution. The Laplace-distributed perturbation is generated by inverse-transform sampling:

(65) $$Z_{\xi }=-\mathrm{sign}\left(U\right)\log\left(1-2|U|\right),\;U∼\mathrm{Uniform}\left(-0.5,0.5\right).$$

Impulsive perturbation is based on Gaussian perturbation and amplifies the perturbation magnitude by a factor of 4 with probability $p_{\mathrm{imp}}=0.08$:

(66) $$Z_{\xi }=\left\{\begin{array}{cc} A, & A_{ij}∼N\left(0,1\right),\\ 4A, & \mathrm{with}\;\mathrm{probability}\;0.08. \end{array}\right.$$

Heavy-tailed perturbation uses a Student-*t* distribution with ν=3 degrees of freedom and variance normalization:

(67) $$Z_{\xi }=\frac{T_{3}}{\sqrt{3}}.$$

The perturbation scales are set to 0.010, 0.010, 0.012, and 0.012 for Gaussian, Laplace-distributed, impulsive, and heavy-tailed perturbations, respectively. All perturbations are independently generated for each sample and each spatial position. After perturbation, matrices are symmetrized again and SPD correction is applied.

**Figure 7:**
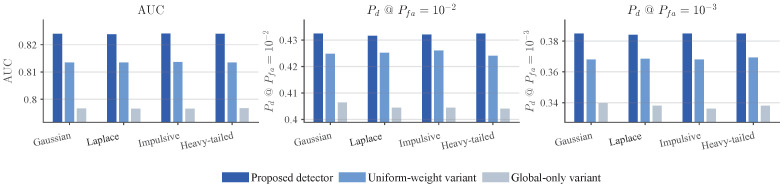
Robustness under non-Gaussian background perturbations.

In this experiment, non-Gaussian perturbations are not applied to the training $H_{0}/H_{1}$ sets. Therefore, the class-conditional reference fields and the discriminative weight map are still estimated from the main-scene training data. Non-Gaussian perturbations are applied only to the calibration $H_{0}$, test $H_{0}$, test $H_{1}$, and audit $H_{0}$ sets. For each noise condition, the fixed-$P_{\mathrm{fa}}$ threshold is re-estimated using the corresponding perturbed independent $H_{0}$ calibration set. Figure 7 reports the robustness results under non-Gaussian background perturbations.

The results indicate that the main performance ordering is not reversed under the four field-level background perturbations. The proposed detector remains higher than the uniform-weight and global-pooling variants in AUC and at both operating points. Since the threshold is recalibrated using the perturbed $H_{0}$ calibration set under each noise condition, this result indicates robustness to moderate field-level distributional perturbations. It does not imply, however, that a threshold calibrated under one noise condition can be directly transferred to another noise condition without recalibration.

## 4. Discussion

### 4.1. Sources of Performance Gain

The experimental results indicate that the primary gain arises from the local SPD-field representation, rather than from the Riemannian metric alone. Compared with the global-energy and pooled-covariance baselines, local structure-field methods preserve the directional organization, anisotropy, and spatial distribution within the patch, and are therefore more suitable for the present local-structure-dominated detection scenario.

Within the local structure-field object layer, AIRM-based comparison and pointwise Karcher mean reference fields provide a geometry-consistent way to define class-conditional reference fields and local distance-difference evidence. However, the empirical gain over the Euclidean/Frobenius structure-field baselines is relatively small. Therefore, the role of AIRM should be understood as a geometry-consistent modeling component within the object-layer framework, rather than as a factor that alone produces a large performance jump.

Discriminative weighting and block-wise robust pooling mainly improve sample-level evidence aggregation. They shift the statistic from a purely global average response toward stable local discriminative regions, thereby reducing the influence of low-information areas, local misalignment, and abnormal responses on the final statistic.

### 4.2. Relationship to Existing Methods and Future Directions

Compared with existing methods, the core difference of the proposed method is not simply the use of a more complex distance or a more complex pooling strategy, but the change in the detection object layer. Classical detection and constant false-alarm rate (CFAR) methods usually operate on scalar statistics and adaptive thresholds under fixed false-alarm probability constraints. They have clear detection-theoretic interpretations, but their ability to represent local structural organization is limited. Spectrogram patch methods preserve time–frequency locality, but most of them still treat patches as intensity templates or learning inputs. Existing matrix information-geometric detectors place covariance matrices or HPD matrices on SPD/HPD manifolds and compare them using intrinsic matrix geometry, forming a mature route for radar detection. However, such methods usually compress each observation cell into a single matrix object. The proposed method lies at the intersection of these routes: it inherits the locality of time–frequency patch methods, transforms local structures into an SPD-valued field through the structure tensor, and performs reference estimation and local distance-difference evidence construction under AIRM. The relationship between the proposed method and representative methodological families is summarized in Table 5.

One representative research line in information-geometric signal detection is the series of works by Cheng, Hua, Wang, Wu, Yang, and collaborators on matrix information geometry detection. This line starts from an information-geometric interpretation of signal detection, connects Neyman–Pearson detection, Kullback–Leibler divergence, and radar CFAR detection, and further develops radar target detection methods based on symmetrized KL divergence, total KL divergence, total Jensen–Bregman divergence, and matrix information geometry. More recent work extends this line to weak target detection in heterogeneous clutter and track-before-detect scenarios in range–azimuth measurements. In particular, discriminative HPD-manifold projection and TBD-MIG detectors further show that manifold projection can enhance the discriminative capability of HPD matrix representations for range-spread radar targets in non-Gaussian clutter [26]. These developments indicate that matrix information geometry has evolved from theoretical interpretation toward more complex radar detection tasks. In contrast to this line, the present work does not continue to construct a detector around a single covariance or HPD matrix object. Instead, it represents each sample as a spatially distributed local SPD structure field in order to preserve the local structural distribution inside a patch.

Another representative line is matrix information geometry for radar processing associated with Barbaresco and related work. This line introduced Cartan/Siegel geometry, SPD/HPD matrix manifolds, Fréchet metric spaces, and related tools into radar covariance matrix processing, and explored robust statistical processing methods such as OS-HDR-CFAR, OS-STAP, and Riemannian means/medians. Along this direction, Ono and Peng recently compared AIRM, Log-Euclidean, and Bures–Wasserstein geometries for HPD matrices in Matrix-CFAR signal detectors, while also analyzing detection performance, outlier robustness, and computational complexity. These developments suggest that an important future direction is not merely to add more detector modules, but to establish a more systematic connection among different SPD/HPD geometries, robust reference estimation, and local structure-field representations.

The additional SPD-manifold studies also help clarify the geometric scope of the present method. General studies of O(n)-invariant metrics emphasize that AIRM is one member of a broader family of meaningful intrinsic geometries on SPD matrices [24]. Riemannian Procrustes analysis further demonstrates that SPD covariance features can be aligned across domains while respecting manifold geometry [25]. These studies mainly operate on covariance, SPD, or HPD matrix objects, whereas the present work focuses on a spatially distributed field of local SPD structure tensors, where the spatial organization of local time–frequency structures is itself part of the discriminative information.

### 4.3. Information-Geometric Interpretation and Its Limits

The design of the proposed method is inspired by a classical relative-closeness decision principle. In classical detection, the likelihood-ratio test can be interpreted, under large-sample conditions, as a comparison between the Kullback–Leibler divergences from the observation distribution to the two hypothesized distributions. This relationship provides an information-geometric motivation for the local distance-difference evidence used in this paper.

However, the proposed detector should not be interpreted as a strict generalization of the classical LRT. The object layer in this paper is the local SPD structure field. The quantity Δ(x) represents local distance-difference evidence under the SPD/AIRM geometry, while discriminative weighting and block-wise robust pooling are sample-level evidence aggregation mechanisms. Under additional approximation assumptions, such as locally isotropic Riemannian Gaussian perturbations, the difference between squared AIRM distances can be monotonically related to an approximate log-likelihood ratio. This relationship should be understood as an explanatory motivation rather than as a strict optimality proof.

### 4.4. Limitations and Applicability

The proposed detector is most suitable for non-stationary detection tasks in which discriminative information is primarily encoded in local structural organization, directional continuity, and spatial arrangement patterns. In such scenarios, the relevant class differences are not adequately captured by global energy, mean spectral intensity, or a single covariance-scale statistic. Conversely, if the class difference is mainly reflected in global energy, average spectral strength, or a single covariance-scale variation, simpler global statistics may already be sufficient, and the advantage of the proposed method may diminish.

A potential class of physical applications is the analysis of measurement signals whose diagnostic information appears as specific local structures in the time–frequency plane. Examples include radar or wireless sensing signals with drifting spectral components, multipath-induced ridges, fragmented time–frequency textures, or local directional continuity. Related GNSS radio-occultation and reflected-signal studies have shown that direct and reflected signal components can produce structured signatures in spectral or time–frequency representations [44,45]. For such problems, the proposed detector may be useful if the class-relevant information can be stably encoded as local ridges, directional organization, or spatially localized texture changes in the time–frequency plane.

However, this applicability is conditional. The present manuscript does not claim direct validation on GNSS radio-occultation data, reflected-signal datasets, or any other specific physical measurement dataset. Applying the proposed method to such problems would require an end-to-end validation pipeline, including raw waveform preprocessing, time–frequency representation design, local SPD structure-field construction, and fixed-$P_{\mathrm{fa}}$ calibration under the corresponding physical clutter or noise conditions. Therefore, validation on real non-stationary physical data is an important future direction rather than a conclusion of the current controlled benchmark.

The benefit of the method also depends on the existence of local discriminative structures that can be stably estimated. When discriminative local structures are too few, overly fragmented, or strongly contaminated by local interference, the gains from discriminative weighting and top-block emphasis may not persist. The boundary setting in the structural perturbation experiment also indicates that the method is not unconditionally superior under all local complexity conditions.

In addition, AIRM distance computation, pointwise Karcher mean estimation, and block-wise pooling introduce higher computational cost than global-energy or pooled-covariance baselines. Therefore, the proposed method is more appropriate for scenarios where structural interpretability and local-structure detection performance are prioritized over minimum-cost detection.

Finally, the experiments in this paper are conducted on a controlled SPD-field object-layer benchmark. These experiments mainly validate the detector mechanism after local structures have been represented as SPD fields. They do not constitute a complete end-to-end validation on raw non-stationary waveforms or real physical measurement datasets. Moreover, the nominal SNR/SCR-like index used in the benchmark is a controlled background-difficulty index and should not be regarded as a measured physical signal-to-noise ratio or signal-to-clutter ratio. End-to-end validation on raw waveforms, real public datasets, cross-scene fixed-$P_{\mathrm{fa}}$ calibration, and more efficient geometric approximations remain important directions for future work.

## 5. Conclusions

This paper has presented an information-geometric detector for non-stationary signal detection problems dominated by local structural differences. The proposed method represents a time–frequency patch as a local SPD structure field, estimates class-conditional pointwise Karcher mean reference fields under AIRM, and constructs local distance-difference evidence from pointwise differences between squared AIRM distances. Through discriminative weighting, block-wise robust pooling, and independent $H_{0}$ calibration, the local distance-difference evidence is integrated into a fixed-$P_{\mathrm{fa}}$ detection statistic.

Controlled SPD-field experiments show that, under the adopted object-layer protocol, the proposed detector achieves the best overall performance among the evaluated methods. The most evident gains are obtained over the global-energy and pooled-covariance baselines, indicating that local SPD structure-field representation is the primary source of improvement. The gains over Euclidean/Frobenius structure-field variants are smaller, suggesting that AIRM mainly acts as a geometry-consistent modeling choice within the same object layer. Structural perturbation and non-Gaussian experiments further indicate that the method remains stable under perturbations around the main scene, but its advantage depends on the presence of local discriminative structures that can be estimated reliably.

## Figures and Tables

**Table 1:** Computational complexity summary of the proposed detector.

Module	Main Operation	Complexity
Training: STFT and structure-field construction	STFT, log-amplitude patch extraction, and pointwise structure-tensor-field construction for all training samples	$O\;\left(N\cdot C_{\mathrm{STFT}}+N\cdot \left|\Omega \right|\right)$
Training: pointwise reference fields	Pointwise estimation of the two Karcher-mean reference fields over Ω	$O\;\left(\left|\Omega \right|\cdot I_{K}\cdot N\right)$
Training: discriminative weighting map	Pointwise computation of Sep(x), ${\mathrm{Disp}}_{0}\left(x\right)$, ${\mathrm{Disp}}_{1}\left(x\right)$, and construction of W(x)	$O\;\left(|\Omega |\cdot N\right)$
Test: structure-field construction	STFT, patch extraction, and structure-tensor construction for the test sample	$O\;\left(C_{\mathrm{STFT}}+\left|\Omega \right|\right)$
Test: local distance-difference evidence	Pointwise computation of $d_{0}\left(x\right)$, $d_{1}\left(x\right)$, and Δ(x)	O(|Ω|)
Test: block-wise robust pooling	Computation of ${\left\{s_{\ell }\right\}}_{\ell =1}^{L}$, sorting, and top-block selection	$O\;\left(\sum_{\ell =1}^{L}\left|B_{\ell }\right|+L\log L\right)$
Test: total online detection	End-to-end computation from the test sample to the final statistic *T*	$O\;\left(C_{\mathrm{STFT}}+\left|\Omega \right|+\sum_{\ell =1}^{L}\left|B_{\ell }\right|+L\log L\right)$
Fixed-$P_{\mathrm{fa}}$ threshold calibration	Repeated statistic evaluation on *M* independent $H_{0}$ calibration samples and empirical quantile extraction	$O\;\left(M\cdot C_{\det}+M\log M\right)$
Memory: detector core	Storage of $S_{0}$, $S_{1}$, W(x), and one test structure field	$O\;\left(|\Omega |\right)$
Memory: training structure fields	Explicit storage of all training structure fields	$O\;\left(N\cdot |\Omega |\right)$

**Table 2:** Core experimental and algorithmic parameter settings.

Category	Parameter	Value	Role
Scene label	Public name	Main structured-locality detection scene	Public scene name
Seeds	Random seeds	101–105	Repeated trials
Main evaluation	Nominal SNR/SCR-like index	−20 dB to 0 dB in 2 dB steps	Nuisance/background difficulty sweep
Fixed-$P_{\mathrm{fa}}$ operating points	$P_{\mathrm{fa}}$	$10^{-2}$, $10^{-3}$	Operating points
Training	Train $H_{0}$/Train $H_{1}$	120/120	Reference-field estimation
Calibration	Calibration $H_{0}$	20,000	Threshold calibration
Testing	Test $H_{0}$/Test $H_{1}$	500/500	AUC and $P_{d}$ evaluation
Audit	Audit $H_{0}$	20,000	Achieved $P_{\mathrm{fa}}$ validation
Patch	Patch size	32×32	Local time–frequency field size
SPD regularity	Minimum eigenvalue	$10^{-4}$	Lower eigenvalue bound
Class structure	Class orientation separation	44°	Class-defining orientation separation
Discriminative jitter	Discriminative rotation jitter	0.4°	Discriminative structure stability
Discriminative perturbation	Discriminative perturbation amplitude	0.006	Local structural perturbation
Local structures	Island radius	3.2	Local structure scale
Local structures	Island amplitudes	1.00, 0.82, 0.68	Discriminative strength
Distractors	Number	6	Nuisance local structures
Distractors	Radius	2.2	Distractor scale
Distractors	Amplitude range	0.22–0.48	Distractor strength
Distractors	Rotation jitter	16°	Distractor variability
Pooling	Block size/stride	8/4	Block-wise evidence pooling
Pooling	Top-block fraction *q*	0.2	Top-block fraction
Pooling	Aggregation coefficient α	0.65	Global/top-block mixing
Weight map	Weight-map smoothing scale	1	Spatial smoothing
Karcher mean	Karcher tolerance/max iterations	$10^{-6}$/30	Reference-field estimation

**Table 3:** Average performance results for baseline comparison under the main scene.

Method	AUC	$P_{d}$ at $10^{-2}$	${\hat{P}}_{\mathrm{fa}}$ at $10^{-2}$	$P_{d}$ at $10^{-3}$	${\hat{P}}_{\mathrm{fa}}$ at $10^{-3}$
Proposed detector	0.8240	0.4316	0.00927	0.3852	0.00111
Euclidean structure-field baseline ^1^	0.8207	0.4252	0.00935	0.3656	0.00098
Template-correlation baseline	0.7580	0.4300	0.01005	0.3732	0.00115
Pooled-covariance baseline	0.7166	0.0436	0.00966	0.0088	0.00089
Global energy baseline	0.5048	0.0116	0.01015	0.0016	0.00118

^1^ The Frobenius structure-field baseline is numerically identical to the Euclidean structure-field baseline in the current implementation and is therefore omitted from the table to avoid duplication.

**Table 4:** Paired performance differences between the proposed detector and competing methods. Positive values indicate an advantage of the proposed detector.

Comparison	Paired Unit	ΔAUC	$\Delta P_{d}@10^{-2}$	$\Delta P_{d}@10^{-3}$
Proposed vs. uniform-weight	seed-avg. sweep (n=5)	0.0118 [0.0101, 0.0134]	0.0192 [0.0113, 0.0271]	0.0159 [−0.0026, 0.0343]
Proposed vs. global-only	seed-avg. sweep (n=5)	0.0238 [0.0169, 0.0306]	0.0392 [0.0272, 0.0513]	0.0448 [0.0193, 0.0703]
Proposed vs. Euclidean/Frobenius SF	seed (n=5)	0.0033 [0.0009, 0.0056]	0.0064 [−0.0005, 0.0133]	0.0196 [−0.0149, 0.0541]
Proposed vs. template correlation	seed (n=5)	0.0660 [0.0405, 0.0915]	0.0016 [−0.0500, 0.0532]	0.0120 [−0.0667, 0.0907]
Proposed vs. pooled covariance	seed (n=5)	0.1073 [0.0793, 0.1353]	0.3880 [0.3712, 0.4048]	0.3764 [0.3366, 0.4162]
Proposed vs. global energy	seed (n=5)	0.3192 [0.2920, 0.3464]	0.4200 [0.3930, 0.4470]	0.3836 [0.3402, 0.4270]

**Table 5:** Relationship between the proposed method and representative methodological families.

Family	Representative Works	Object	Main Advantage	Difference from This Work
Classical/CFAR detection	Kay; Finn and Johnson; Rohling; Kelly [1,40,41,42]	Scalar statistic/adaptive threshold	Clear fixed-$P_{\mathrm{fa}}$ interpretation	Weak local-structure modeling
Spectrogram patch methods	Sahni; Espi; Espi [4,5,6]	Spectrogram patches	Preserve time–frequency locality	Lack SPD geometric modeling
Structure-tensor methods	Bigun; Weickert; Brox [7,8,9]	Local second-order tensor	Encode orientation and anisotropy	Usually not formulated as detectors
Riemannian covariance classification	Barachant; Barachant [15,16]	Covariance matrix	Mature SPD geometry	Mainly classification-oriented
Matrix information-geometric detectors	Barbaresco; Cheng; Hua; Ono and Peng; Yang [17,18,19,20,22,23,26]	Covariance/HPD matrix	Intrinsic matrix geometry	Mostly single-matrix objects
Proposed method	This paper	Local SPD structure field	Preserves locality and SPD geometry	Higher cost; requires stable local structures

## Data Availability

The data used in this study are procedurally generated controlled SPD-field samples. No external dataset was used in the main experiments. The source code, scene-generation scripts, configuration files, random seeds, and per-run CSV result files supporting the reported figures and tables are publicly available at https://github.com/YaohaoYue-79/local-spd-structure-field-detection.git (accessed on 1 June 2026). Since the experimental samples are procedurally generated, the complete experimental data can be regenerated from the released scripts and random seeds.

## Abbreviations

The following abbreviations are used in this manuscript:

AIRM	Affine-invariant Riemannian metric
AUC	Area under the receiver operating characteristic curve
CFAR	Constant false-alarm rate
HPD	Hermitian positive definite
KL	Kullback–Leibler
LRT	Likelihood-ratio test
ROC	Receiver operating characteristic
SCR	Signal-to-clutter ratio
SNR	Signal-to-noise ratio
SPD	Symmetric positive definite
STFT	Short-time Fourier transform
